# Mitigation of *P. gingivalis*-exacerbated intestinal inflammation by paeoniflorin through alteration of the gut microbiota

**DOI:** 10.1128/spectrum.00002-24

**Published:** 2025-06-26

**Authors:** Ru Qu, Ming Li, Ping Li, Ying Song, Biao Dong, Juan Liu, Xuan Mo, Zhenjiang Zech Xu, Xiaochang Huang

**Affiliations:** 1 State Key Laboratory of Food Science and Resources, Nanchang University 547468 , Nanchang, China; 2 Institute of Biological Resources, Jiangxi Academy of Sciences 610487 , Nanchang, China; University of California, Davis, Davis, California, USA

**Keywords:** paeoniflorin, *Porphyromonas gingivalis*, gut microbiota, metabolomic, intestinal inflammation, gut metabolism

## Abstract

*Porphyromonas gingivalis* is a well-known periodontal pathogen that has also been linked to systemic diseases. In this study, our results revealed that *P. gingivalis* exacerbated inflammation, as indicated by weight loss, decreased colon length, and increased colonic tissue inflammation. Furthermore, *P. gingivalis* increased the population of Th17 cells in the spleen and upregulated the expression of pro-inflammatory cytokines in mice. Additionally, *P. gingivalis* aggravated the gut microbiota dysbiosis by reducing the beneficial bacteria like S24-7. In addition, we discovered that paeoniflorin, a widely recognized biologically active phytochemical, had the potential to mitigate the exacerbated inflammation caused by *P. gingivalis*. Specifically, paeoniflorin treatment rebalanced immune cell populations, reduced the levels of inflammatory cytokines, and reshaped the composition of the gut microbiota. In summary, our findings suggest that paeoniflorin holds promise as a potential therapeutic agent for alleviating the inflammatory effects induced by *P. gingivalis*.

**IMPORTANCE:**

*Porphyromonas gingivalis* may reach the gastrointestinal tract and establish colonization. By investigating its effects on mice with mild intestinal inflammation, this study highlights a concerning link between oral pathogens and broader health implications. *P. gingivalis* not only exacerbates inflammation, causing weight loss and colonic tissue damage, but also induces significant changes in the gut microbiota. The identification of paeoniflorin as a potential therapeutic agent adds a promising dimension to addressing these issues. Paeoniflorin shows promise in rebalancing immune responses, reducing inflammation, and reshaping the gut microbiota. This study not only deepens our understanding of the intricate connections between oral and systemic health but also suggests a potential avenue for developing therapeutic strategies to mitigate the impact of *P. gingivalis* on overall well-being.

## INTRODUCTION

*Porphyromonas gingivalis* (Pg) is a gram-negative bacterium and a worldwide pathobiont of the human oral cavity (1, 2). *P. gingivalis* inhibits innate and adaptive immune responses by secreting virulence factors, including fimbriae, gingipains, and lipopolysaccharide, which promote its survival and propagation in the host (3, 4). Beyond its traditional habitat in the oral cavity, *P. gingivalis* has been identified in systemic sites, such as atherosclerotic plaques, synovial fluid, and the gastrointestinal tract. This expanded distribution underscores the pressing need to explore its potential impact on systemic health. *P. gingivalis* is closely related not only to oral diseases (5) but also to non-oral diseases such as Alzheimer’s disease (6), cardiovascular disease (7, 8), rheumatoid arthritis (9), and inflammatory bowel disease (IBD) (10, 11). The underlying mechanisms may result from the transmission of oral microbes to the gastrointestinal system and other distant sites probably through processes like salivary swallowing and bloodstream dissemination (12).

Previous studies have suggested that intestinal colonization of *P. gingivalis* is associated with gut inflammation. Tsuzuno et al*.* showed that oral administration of *P. gingivalis* can disrupt the intestinal epithelial barrier by reducing the levels of tight junction protein in mice, significantly exacerbating colitis (13). In another study, *P. gingivalis* was intrarectally implanted into mice with chemically induced colitis (14), which resulted in a significant aggravation of disease activity index (DAI) score, accompanied by increased colon epithelial loss and heightened inflammation cell infiltration (14). Furthermore, it has been observed that *P. gingivalis* can also indirectly induce intestinal inflammation by modulating the gut microbiota and disrupting epithelial barrier function (15). These experimental investigations underscore the multifaceted impact of *P. gingivalis* in the context of intestinal inflammation.

Additionally, we aimed to explore potential therapeutic strategies that could ameliorate the inflammatory effects of *P. gingivalis*. Paeoniflorin is a monoterpene glucoside, a natural compound from *Paeonia lactiflora Pall*. Paeoniflorin, a known bioactive compound, possesses strong anti-inflammatory and immune regulatory effects. Related research has reported that paeoniflorin decreased the infiltration of gram-positive bacteria in intestines to alleviate mice colitis (16). Furthermore, paeoniflorin may regulate the renewal and differentiation of intestinal stem cells to promote the regeneration and repair of intestinal epithelium, thus improving mucosal damage (17).

In this study, we examined *P. gingivalis’s* impact on mild intestinal inflammation in mice. *P. gingivalis* exacerbated inflammation, causing weight loss, colon shortening, and colonic inflammation. It also elevated Th17 cells, increased pro-inflammatory cytokines, and aggravated gut microbiota dysbiosis. Paeoniflorin mitigated these effects, restoring immune balance and reshaping the gut microbiota. Paeoniflorin holds promise as a therapeutic agent for intestinal inflammation, offering a potential strategy for preventing or treating oral bacterial translocation and the associated progression of related diseases.

## MATERIALS AND METHODS

### Microbial culture conditions

*P. gingivalis* strain W83 was obtained from the Guangdong Microbial Culture Collection Center (GDMCC). *P. gingivalis* was cultured in brain heart infusion broth with 5 mg/L hemin and 0.5 mg/L vitamin K. Bacterial culture was performed under strict anaerobic conditions (10% H_2_, 10% CO_2_, and 80% N_2_) at 37°C.

### Animal treatment

Female C57BL/6J mice (aged 6–8 weeks) were purchased from Hunan Silaike Laboratory Animal Corporation Ltd. (Changsha, China, license number: SCXK [Xiang] 2019-0004]. The mice were fed a commercial rodent chow (Beijing Vital River Laboratory Animal Technology Corporation Ltd.) and deionized water *ad libitum*. The mice were kept in individually ventilated cage systems at 22 ± 2°C and 50 ± 5% relative humidity with a 12:12 h light-dark cycle in specific-pathogen-free conditions.

**Fig 1:**
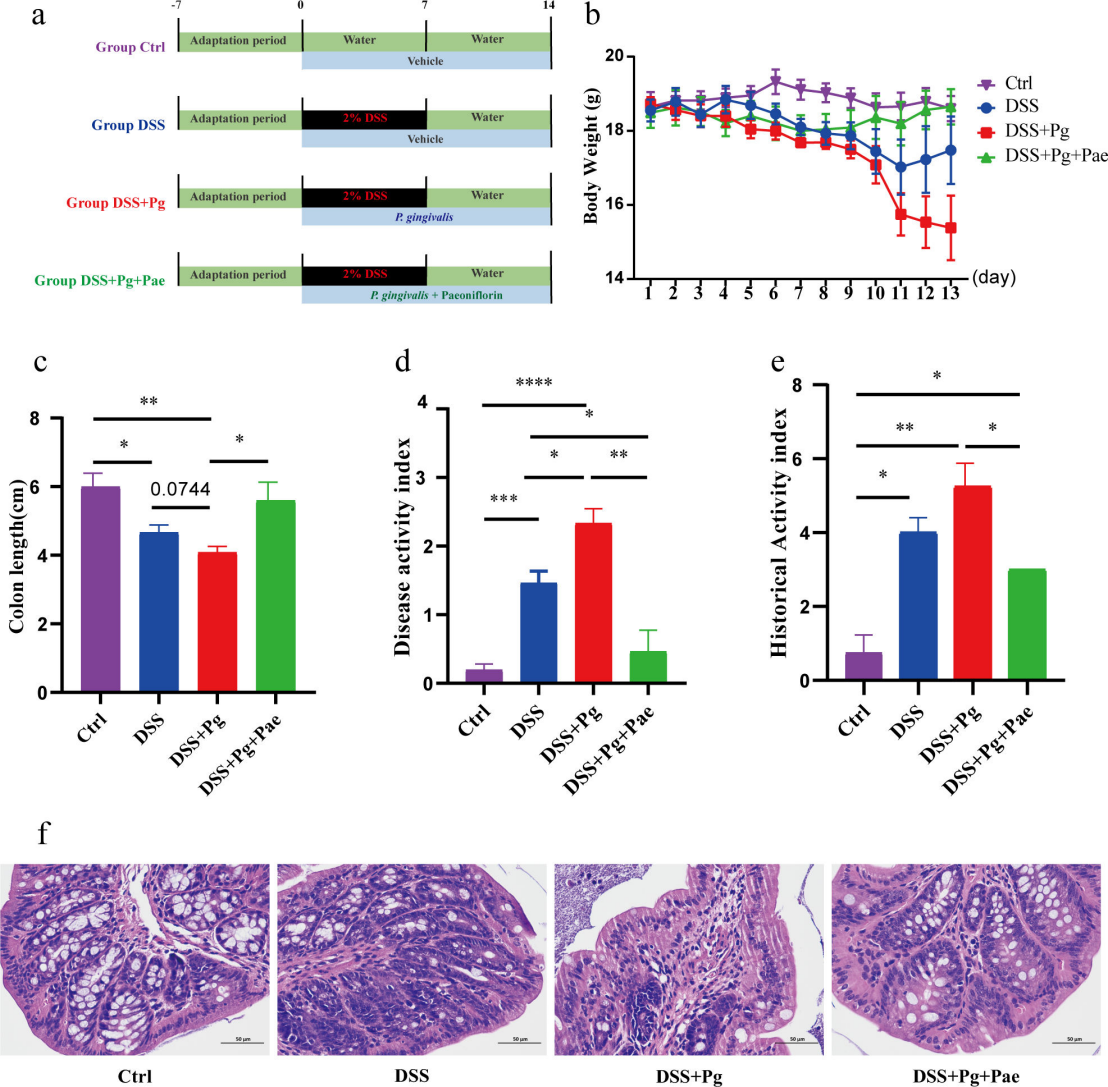
*P. gingivalis* exacerbates intestinal inflammation, a process ameliorated by paeoniflorin. (**a**) Experimental design. (**b**) Body weight. (**c**) Colon length. (**d**) DAI. (e and f) Hematoxylin and eosin (H&E) staining of colon tissue, scale bar: 50 µm. *P < 0.05, **P < 0.01, ***P < 0.001, ****P < 0.0001.

As shown in Fig. 1a, after 1 week of acclimatization, the mice were randomly divided into four groups (*n* = 6 per group): control group (Ctrl), dextran sulfate sodium (DSS) group (DSS), DSS + *P. gingivalis* group (DSS + Pg), and DSS + *P. gingivalis* + paeoniflorin group (DSS + Pg + Pae). Except for the Ctrl group, all other groups were given drinking water containing 2% (wt/vol) DSS (MW: 36,000–50,000, MP Biomedicals, USA) for 7 days to induce intestinal inflammation. Mice requiring oral administration of Pg were given a daily dose of 10^8^ CFUs of live *P. gingivalis* W83 starting from day 1. The mice of the DSS + Pg + Pae group were given paeoniflorin (Aladdin, Shanghai, China) at a dose of 200 mg/kg per day (18) for 14 days.

### Assessment of the severity of colitis by measuring the DAI and histological activity index (HAI)

To assess the DAI score in mouse colon, mice were monitored for body weight loss, stool consistency, and rectal bleeding. Body weight loss was recorded as a percentage of the initial weight, stool consistency was scored from 0 (normal) to 4 (diarrhea), and rectal bleeding was scored from 0 (no blood) to 4 (gross bleeding). The DAI was calculated by summing the scores of these three parameters and averaging them to provide an overall measure of disease severity.

Colon segments were harvested, fixed in 4% paraformaldehyde, dehydrated with ethanol, and embedded in paraffin. Paraffin sections were deparaffinized, rehydrated, and stained with hematoxylin and eosin (H&E) for histological analyses and calculating the HAI score (19).

### Splenocyte culture *in vitro* and cytokine analysis

The spleen was smashed and passed through 70 µm filters to generate a single-cell suspension. The suspension was lysed for 4 min by erythrocyte lysate, after which the supernatant was abandoned by centrifugation at 1,500 × *g* for 8 min. The pelleted cells were resuspended in RPMI 1640 and transferred to a complete medium (RPMI 1640 with 10% fetal bovine serum [FBS]). Cultured CD4^+ ^T cells were first stimulated with cell activation cocktail (BioLegend) for 6 h in 5% CO_2_ at 37°C. Subsequently, the cells were stained with fixable viability stain 700 (564997, BD Biosciences) for 30 min in the dark. Then, anti-CD4 (566408, BD Biosciences) and anti-CD25 (564458, BD Biosciences) were added for staining for 30 min at 4°C in the dark, and the cells were fixed and permeabilized with fixation/permeabilization working solution (BioLegend) and permeabilization buffer for 20 min at 4°C in the dark. Finally, the cells were stained with anti-IL-17A (561020, BD Biosciences) and anti-Foxp3 (560402, BD Biosciences) antibodies. FlowJo software was used for data analysis.

### Enzyme-linked immunosorbent assay

The levels of interleukin-6 (IL-6) and IL-17A were measured by using the enzyme-linked immunosorbent assay (ELISA) kit (Thermo Scientific) according to the manufacturer’s instructions.

### Quantitative real-time PCR

Total RNA was extracted from colonic samples homogenized in RNAiso Plus (Takara, China). RNA was used to generate cDNA with PrimeScript RT reagent kit (Takara, China). The resulting cDNA was then subjected to qPCR using TB Green Premix Ex Taq II (Takara, China) on a CFX Connect real-time PCR detection system (Bio-Rad Laboratories, Singapore). The experiment was conducted following the procedures outlined in previously published work (20). The relative mRNA expression levels were determined with the 2^−ΔΔCt^ method with the housekeeping glyceraldehyde-3-phosphate dehydrogenase (GAPDH) gene as the internal reference control.

### DNA extraction and 16S rRNA gene sequencing

The genomic DNA was extracted from feces samples using a magnetic soil and stool DNA kit (Tiangen, Beijing, China). The concentration of DNA was assessed using Nanodrop (Thermo Fisher Scientific, Santa Clara, CA, USA).

The V4 region of bacterial 16S rRNA was selected for analysis using Illumina MiSeq sequencing, which was carried out by a commercial company (Novogene Co., Ltd., Beijing, China). Sequencing libraries were prepared following the manufacturer’s recommendations, using the TruSeq DNA PCR-free sample preparation kit, with the addition of index codes. Library quality was assessed using the Qubit 2.0 Fluorometer (Thermo Scientific, MA, USA) and the Agilent Bioanalyzer 2100 system (Agilent Technologies, CA, USA). Subsequently, the library was subjected to sequencing on an Illumina NovaSeq platform, which generated 250 bp paired-end reads. Analyses of the 16S rRNA gene sequences were performed with Quantitative Insights into Microbial Ecology version 2 (QIIME2, version 2022.8) (21–23). Multiplexed single-end sequencing reads were imported into the QIIME2 platform. Raw reads were quality filtered, assembled, and chimeric sequences were removed using data2, which generated unique amplicon sequence variants. Determinations of alpha and beta diversities were also conducted in QIIME 2.

### Untargeted metabolomics analysis

Metabolites in cecal content samples were determined using high-performance liquid chromatography-quadrupole time-of-flight mass spectrometry (HPLC-Q-TOF/MS, X500R, SCIEX). Approximately 50 mg of cecal content sample was homogenized with 200 µL of ddH_2_O and mixed with 800 µL of methanol:acetonitrile (1:1). The mixture was incubated at 20°C for 1 h, then centrifuged at 12,000 rpm and 4°C for 15 min to pellet proteins, and the supernatant was dried. Subsequently, the dry sample was dissolved in 200 µL of a solution (acetonitrile:water, 1:1), the supernatant was collected by centrifugation at 12,000 rpm and 4°C for 15 min, and the sample was subjected to liquid chromatography-mass spectrometry analysis. The analyses were performed using a spectrophotometric method.

Preparation of quality control (QC) samples: 10 µL from each sample in each group was equally aspirated, mixed thoroughly, and placed in an autosampler vial for testing. Before performing the formal sample analysis using HPLC-Q-TOF/MS, three consecutive QC samples were run to equilibrate and stabilize the instrument system. During formal sample analysis, one QC sample was run for every five samples to ensure the stability of the instrument analysis system. Detection was started in negative mode, followed by the positive mode.

Using the ProteoWizard software, the raw data generated from HPLC-Q-TOF/MS was converted into the .mzXML format. Subsequently, the “xcms” program from the R package was employed for peak detection, peak filtering, and peak alignment, resulting in a multidimensional data matrix encompassing *m*/*z* values, retention times, and peak areas. This matrix was then subjected to metabolite identification using the MetDNA platform. Employing MetaboAnalyst 5.0 (24), the identified metabolites were subjected to analysis, incorporating various normalization steps such as filtering, logarithmic transformation, and scaling. Ultimately, differential metabolite analysis was conducted, facilitating the exploration of variations within the data set. The screening of differential metabolites mainly referred to variable importance projection (VIP), fold change (FC), and *P* value.

### Statistical analysis

Unless otherwise stated in the individual methods sections above, all the statistical analyses were performed with GraphPad Prism 9.0 software (La Jolla, CA, USA). Comparisons between the two groups were assessed by two-tailed Student’s *t*-test. *P* value <0.05 was considered to be statistically significant in all statistical analyses. The numbers of animals (*n*) used for individual experiments and details of the statistical tests used are indicated in the respective figure legends.

## RESULTS

### *P. gingivalis* exacerbates intestinal inflammation, a process ameliorated by paeoniflorin

The administration of *P. gingivalis* led to a notable exacerbation of weight loss in the mice, as depicted in Fig. 1b. Oral administration of *P. gingivalis* aggravated intestinal inflammation in mice, leading to a considerable reduction in colon length (Fig. 1c). For a more comprehensive assessment of colitis severity, the DAI score was assessed by loss of weight, stool consistency, and the presence of blood in stool. The DAI score exhibited a significant increase following *P. gingivalis* administration (Fig. 1d). In the DSS group, histopathological evaluation revealed partial inflammatory cell infiltration and disruption of mucosal epithelial integrity. The DSS + Pg group displayed more severe damage, characterized by the destruction of the crypt structure and the increase of inflammatory infiltration (Fig. 1e and f). Taken together, *P. gingivalis* exacerbates enteritis-related symptoms in mice, including weight loss, reduced colon length, elevated DAI score, and substantial damage to colon integrity.

Following the administration of paeoniflorin to the DSS + Pg treated mice, we observed a reduction in weight loss, colon shortening, and the DAI score (Fig. 1b through d). H&E staining further supported the efficacy of paeoniflorin treatment, which demonstrated a significant attenuation of severe histopathological damage (Fig. 1e and f). These findings indicate that paeoniflorin treatment effectively alleviates the inflammatory symptoms exacerbated by *P. gingivalis*.

### *P. gingivalis* and paeoniflorin shifted Th17/Treg balance

**Fig 2:**
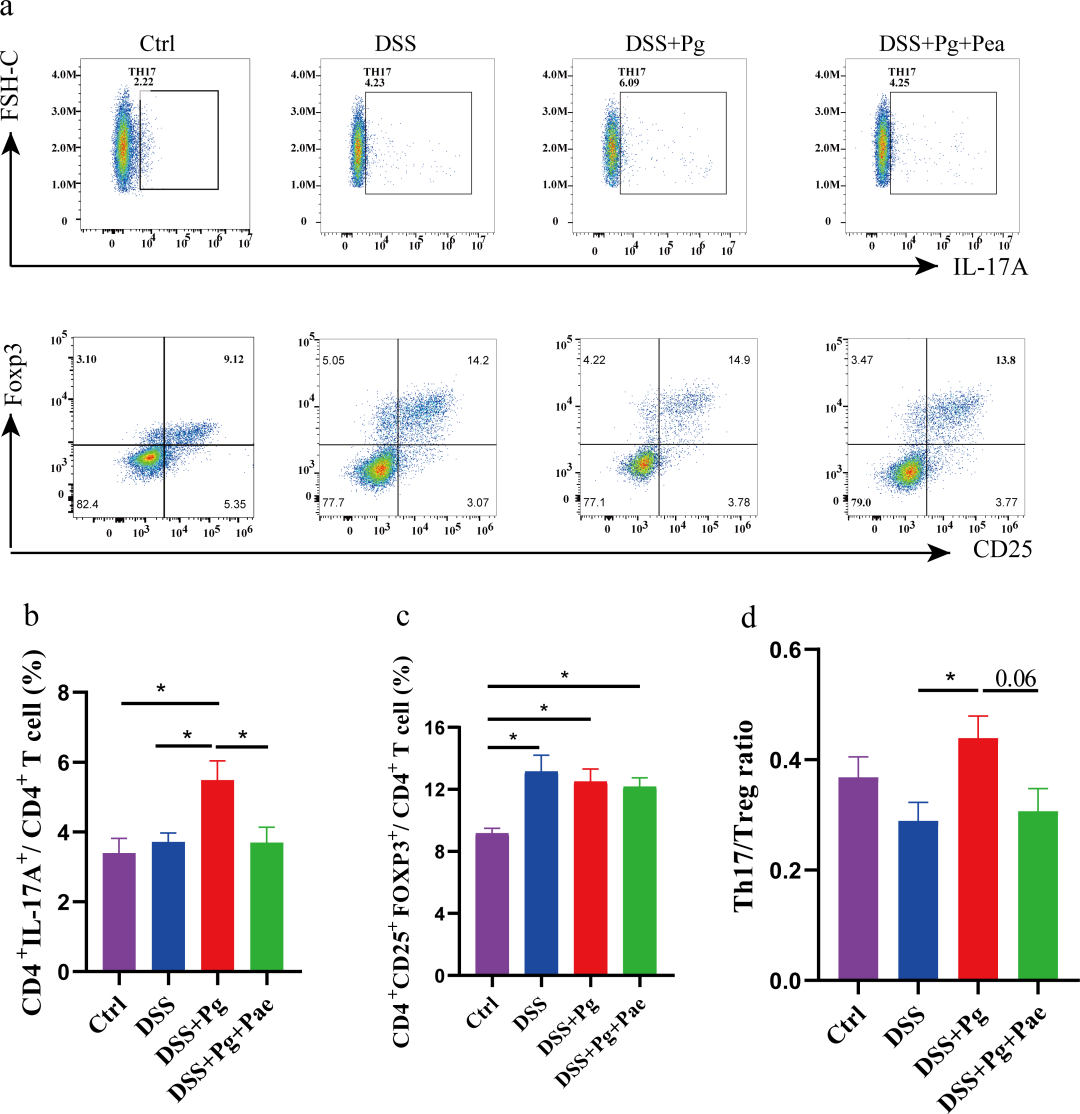
*P. gingivalis* and paeoniflorin shifted Th17/Treg balance. (**a**) Mouse spleen cells were collected, and the frequency of CD4^+^IL-17A^+^ Th17 cells and CD4^+^CD25^+^Foxp3^+^ Treg cells were examined by flow cytometry. (**b**) The frequency of Th17 cells in the spleen. (**c**) The frequency of Treg cells in the spleen. (**d**) The Th17/Treg cell ratio in the spleen. *P < 0.05, **P < 0.01.

The balance between Th17 cells and Treg cells is widely recognized as pivotal in the context of autoimmune and inflammatory responses within the gut. In this study, we used flow cytometry to assess the dynamics of Th17 and Treg cells within murine splenic tissues (Fig. 2a).

Compared to the Ctrl group, the other three groups had a higher proportion of Treg cells, suggesting a typical immune response in mice with inflammation. We observed a significant increase in the proportion of Th17 cells in mice administered with *P. gingivalis* compared to the DSS group (Fig. 2b). The level of Treg cells did not show a significant change (Fig. 2c), while the Th17/Treg cell ratio was markedly increased (Fig. 2d). Conversely, paeoniflorin intervention resulted in a reduction in the level of Th17 cells (Fig. 2b), with no significant changes observed in the Treg population (Fig. 2c). The Th17/Treg cell ratio was restored by paeoniflorin intervention (Fig. 2d). These findings underscore the disruptive role of *P. gingivalis* in upsetting the equilibrium between Th17 and Treg cells, which contributes to heightened gut inflammation. Paeoniflorin has the potential to restore the normal gut status from these disruptions and immunological imbalance.

### *P. gingivalis* and paeoniflorin modulate colitis-associated inflammatory factors

**Fig 3:**
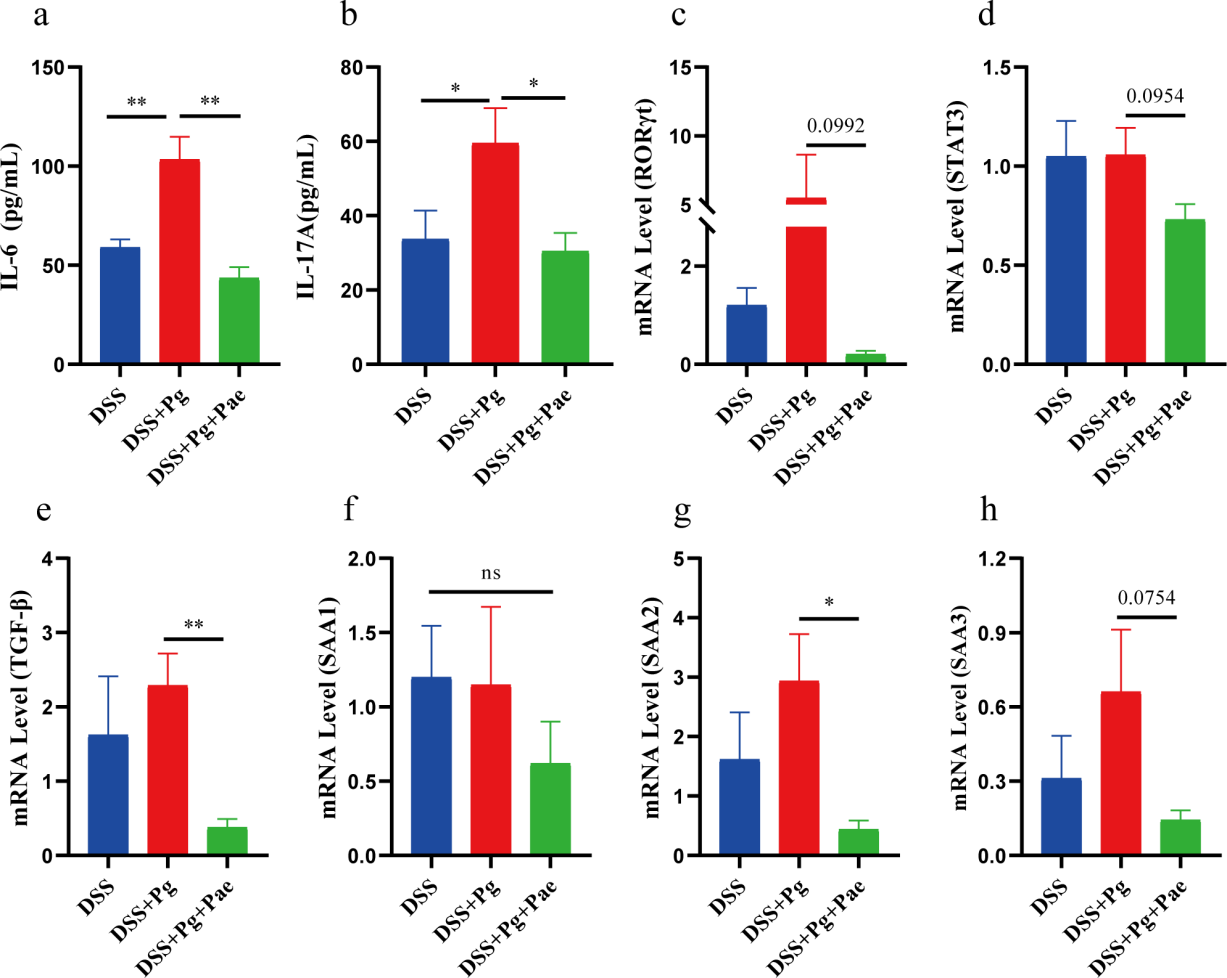
*P. gingivalis* and paeoniflorin modulate colitis-associated inflammatory factors. The level of IL-6 in serum (**a**) and IL-17A in colon tissues (**b**). (c–h) The mRNA levels of ROR-γT, STAT3, TGF-β, SAA1, SAA2, and SAA3. *P < 0.05, **P < 0.01.

Previous research has demonstrated the essential role of ROR-γT in driving the differentiation of CD4 T cells into Th17 cells and promoting IL-17 expression (25, 26). We noticed an upward trend in the mRNA expression of ROR-γT within the colon tissue of the DSS + Pg group (Fig. 3c). IL-17 is primarily produced by Th17 cells and plays a significant role in a variety of immune responses. We utilized an ELISA kit to measure the levels of IL-17A in mouse colonic supernatant and found that *P. gingivalis* significantly promoted an increase in IL-17A levels (Fig. 3a). IL-6 is a cytokine with both pro-inflammatory and anti-inflammatory roles, crucial for regulating immune responses. We found significantly elevated IL-6 levels in the serum of DSS + Pg group (Fig. 3b). qPCR analysis showed that STAT3 expression in colon tissue was similar in the DSS + Pg and DSS groups (Fig. 3d). These results suggest that *P. gingivalis* may worsen intestinal inflammation by disrupting immune homeostasis. After paeoniflorin treatment, IL-17A and IL-6 levels decreased significantly (Fig. 3a and b), and ROR-γT and STAT3 expression were lower in the paeoniflorin group compared to the DSS + Pg group, though not statistically significant (Fig. 3d). Additionally, paeoniflorin treatment significantly reduced mRNA level of transforming growth factor-β (TGF-β) compared to the DSS + Pg group (Fig. 3e). Thus, we hypothesize that paeoniflorin alleviates intestinal inflammation by inhibiting the IL-6/STAT3/IL-17 pathway, although further experiments are needed to confirm this hypothesis.

Furthermore, serum amyloid A (SAA) is a highly conserved family of acute-phase response proteins and essential in the immune-mediated inflammatory process (27). Previous studies have shown that SAAs can induce pathogenic Th17 cells and contribute to inflammatory diseases (28, 29). Therefore, we assessed the mRNA levels of SAAs in colon tissues (Fig. 3f through h) and observed elevated SAA2 and SAA3 in both the DSS group and the DSS + Pg group, with no significant differences between them. Following paeoniflorin intervention, there was a noticeable reduction in the levels of both SAA2 and SAA3. SAA1 level did not exhibit a significant change. These findings collectively demonstrate that paeoniflorin and *P. gingivalis* modulate inflammatory factors in colitis, presenting promising avenues for further research and therapeutic interventions.

### Paeoniflorin restored the gut microbiota dysbiosis caused by *P. gingivalis*

**Fig 4:**
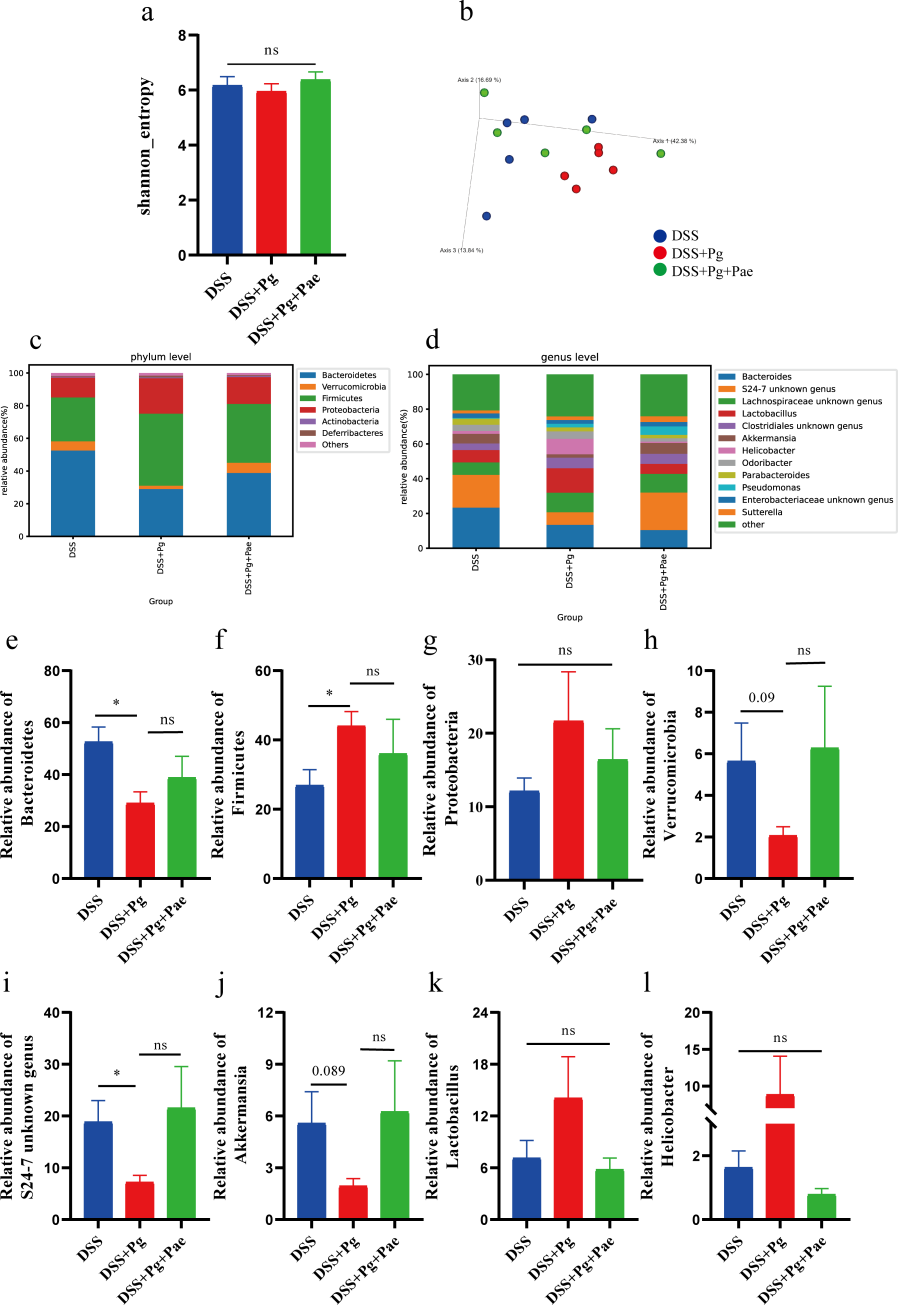
Paeoniflorin restored the gut microbiota dysbiosis caused by *P. gingivalis*. (**a**) Alpha diversity analysis of Shannon index. (**b**) Principal coordinate analysis plot based on weighted UniFrac distance. (c and d) Microbiota composition at the phylum (**c**) and genus (**d**) level. (e–h) The relative abundance of Firmicutes (**e**), Bacteroidetes (**f**), Proteobacteria (**g**), and Verrucomicrobia (**h**). (i–l) The relative abundance of S24-7 unknown genus (**i**), *Akkermansia* (**j**), *Lactobacillus* (**k**), and *Helicobacter* (**l**). *P < 0.05.

The regulatory effect of paeoniflorin on the gut microbiota was further studied by 16S rRNA amplicon sequencing. There was no significant change in alpha diversity among the different treatment groups (Fig. 4a). Principal coordinate analysis based on weighted UniFrac distance revealed that gut microbiota from the DSS + Pg group formed a distinct cluster separate from the DSS group, indicating significant changes in microbiota composition induced by *P. gingivalis* (Fig. 4b). The DSS + Pg + Pae group also formed a distinct cluster separate from the DSS + Pg group, with the gut microbiota of Pae-treated mice showing a tendency to shift back toward the DSS group. This suggests that paeoniflorin can modulate Pg-induced dysbiosis.

At the phylum level (Fig. 4c), *P. gingivalis* reduced the abundance of Bacteroidetes significantly and increased the abundance of Firmicutes. Paeoniflorin increased the abundance of Bacteroidetes while decreasing the abundance of Firmicutes (Fig. 4e and f). The average relative abundance of Proteobacteria was highest in the DSS + Pg group and decreased after paeoniflorin treatment, although these changes were not statistically significant (Fig. 4g). Verrucomicrobia aids in glucose homeostasis and has anti-inflammatory properties beneficial for gut health (30). Its abundance was the lowest in the DSS + Pg group and appeared to increase after paeoniflorin intervention but not exactly statistically signiﬁcant (Fig. 4h).

At the genus level, S24-7 exhibited a significant decrease in abundance following *P. gingivalis* administration (Fig. 4i) and an increase after paeoniflorin treatment, although statistical significance was not reached (Fig. 4i). Similarly, the abundance of *Akkermansia*, which is considered a next-generation probiotic, was reduced in the DSS + Pg group, followed by an increase after paeoniflorin treatment (Fig. 4j). Besides, we observed that *P. gingivalis* increased the relative abundance of *Lactobacillus* and *Helicobacter*, and paeoniflorin had the potential to modulate these changes, despite no statistical signiﬁcance (Fig. 4k and l).

These findings underscore the preventive effects of paeoniflorin on gut microbiota dysbiosis induced by *P. gingivalis,* with potential implications for the broader understanding of gut health and therapeutic interventions.

### Influence of *P. gingivalis* and paeoniflorin on gut metabolism in inflammatory mice

In addition to regulating the gut microbiota, metabolic changes are a significant hallmark of enteritis. Therefore, we employed an untargeted fecal metabolomics analysis to investigate the effects of *P. gingivalis* and paeoniflorin on gut metabolism.

**Fig 5:**
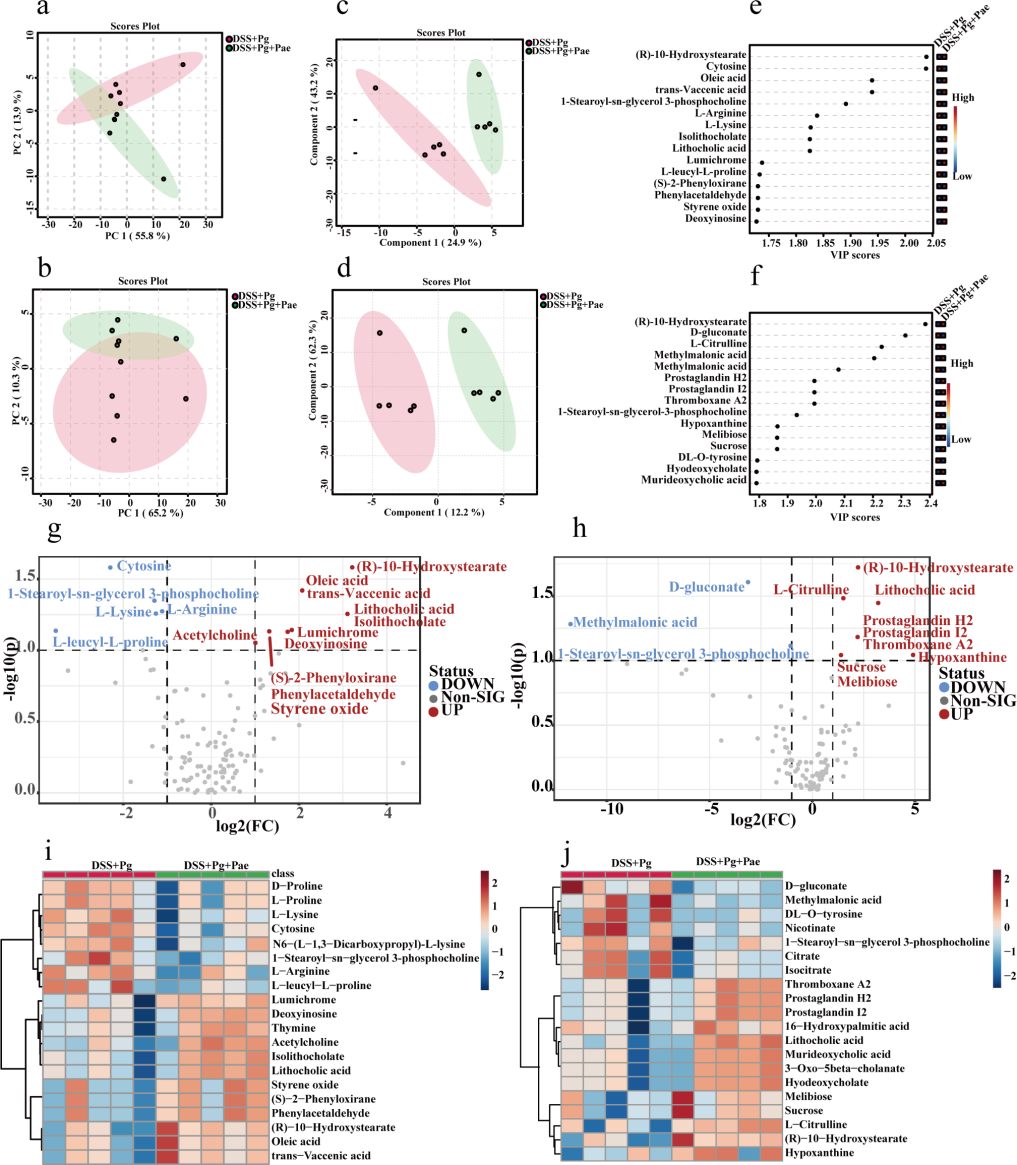
Influence of *P. gingivalis* and paeoniflorin on gut metabolism in inflammatory mice. (a and b) The PCA score plot in the positive and negative ion modes of mass spectrometry, respectively. (c and d) The PLS-DA score plot in the positive and negative ion modes, respectively. (e and f) VIP value chart of the top 15 differential metabolites in the positive and negative modes, respectively. (g and h) Volcano plot between two groups in the positive and negative ion modes, respectively. (i and j) Heat map of top 20 differential metabolites in positive and negative modes, respectively (*t*-test).

Principal component analysis (PCA) and partial least squares-discriminant analysis (PLS-DA) provided complementary insights, with PCA showing overall variance and groupings, while PLS-DA highlighted specific treatment differences. As shown in Fig. 5a through d, DSS + Pg and DSS + Pg + Pae samples clustered separately in both analyses, indicating that paeoniflorin treatment altered the gut metabolome in the DSS + Pg group. Differential metabolites were identified based on VIP scores, with 63 in positive mode and 45 in negative mode (Fig. 5e and f).

Further screening based on FC (≥2) and *P* values (≤0.1) revealed significant changes. In positive mode, 11 metabolites, including phenylacetaldehyde and lithocholic acid, were upregulated, while five metabolites, such as L-arginine, were downregulated (Fig. 5g). In negative mode, nine metabolites, including L-citrulline and prostaglandins, showed upregulation, whereas three metabolites, including D-gluconate, were downregulated (Fig. 5h through j).

### Pathway analysis and Spearman correlation analysis heatmap

**Fig 6:**
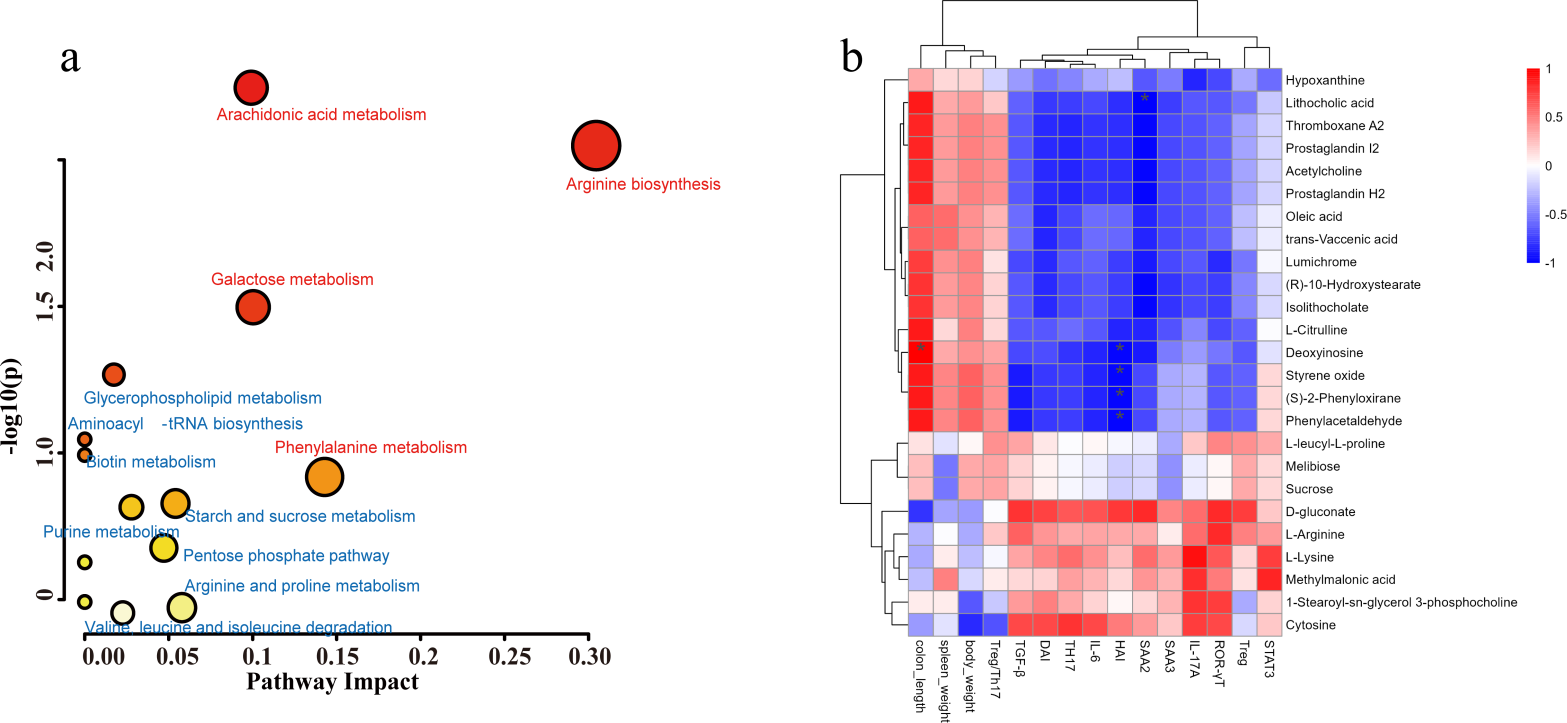
Pathway analysis and Spearman correlation analysis heatmap. (a) Kyoto Encyclopedia of Genes and Genomes pathway analysis of differential metabolites. Pathways with an impact value ≥0.1 are highlighted in red. (b) The color scale indicates the correlation analysis value. The deeper red or blue indicates higher correlation. Grids in red indicate positive correlations, while grids in blue indicate negative correlations. *P < 0.05.

Metabolic pathway enrichment analysis using MetaboAnalyst (24) identified significant enrichment in pathways such as arginine biosynthesis, phenylalanine metabolism, lactose metabolism, and arachidonic acid metabolism (Fig. 6a). This untargeted metabolomic analysis provides a further view of the intricate metabolic changes occurring in mild colitis after treatment with paeoniflorin and *P. gingivalis*.

Spearman correlation analysis between fecal differential metabolites and phenotypes revealed significant associations between the majority of metabolites and inflammatory biomarkers (Fig. 6b). Notably, metabolites such as hypoxanthine, lithocholic acid, thromboxane A2, prostaglandin E2, acetylcholine, and prostaglandin H2 displayed negative correlations with DAI scores, IL-6 levels, SAA2 expression, and the proportion of Th17 cells (Fig. 5i, j and Fig. 6). These metabolites were found to be enriched in the DSS + Pg + Pae group. Conversely, metabolites like L-leucyl-L-proline, melibiose, sucrose, D-gluconate, L-arginine, L-lysine, methylmalonic acid, 1-stearoyl-sn-glycerol 3-phosphocholine, and cytosine exhibited positive correlations with inflammatory indicators, and they were more abundant in the DSS + Pg group (Fig. 5i, j and Fig. 6b).

## DISCUSSION

As a pivotal periodontal pathogenic bacterium, *P. gingivalis* plays a crucial role in the onset and progression of periodontitis. An increasing body of research suggests that periodontal pathogens can migrate to other sites in the body, leading to more severe diseases and becoming risk factors for various health conditions. The disruption of the intestinal barrier function can increase the penetration and invasion of pathogens, resulting in heightened intestinal inflammation. In our study, we observed that mild inflammation induced by DSS resulted in colonic tissue damage, which favored the colonization of the exogenous pathogenic bacterium *P. gingivalis. P. gingivalis* exacerbated inflammatory cell infiltration and disrupted intestinal mucosal epithelial integrity.

Biologically active phytochemicals have been discovered in the field of medicine to enhance intestinal immune function. Several reports showed that paeoniflorin modulates the functions and activation of immune cells, decreases inflammatory medium production, and restores abnormal signal pathways. In this study, after treatment with paeoniflorin, mice exhibited significant downregulation of pro-inflammatory factors. Additionally, the expression of phosphorylation-related protein STAT3 was reduced. Previous studies have also shown that paeoniflorin can reduce the expression of IL-6 (31), IL-17 (32), and STAT3 (33) in pathology. Paeoniflorin also led to a significant decrease in Th17 cells and the restoration of the Th17/Treg cell ratio. Paeoniflorin alleviates inflammation and tissue damage in the mouse intestine by reducing Th17 cell infiltration, suppressing the secretion of pro-inflammatory cytokines, and potentially inhibiting the pathogenicity of *P. gingivalis*.

The gut microbiome plays a critical role in establishing a well-balanced immune system and maintaining immune homeostasis. Studies have found that *P. gingivalis* can alter intestinal composition to some extent. Gavage of *P. gingivalis* in arthritis mice caused changes in gut microbiota and systemic inflammation (34). In our study, we conducted 16S rRNA sequencing and found that *P. gingivalis* had a significant effect on Bacteroidetes and Firmicutes in mice, in line with previous studies (34). After administering paeoniflorin treatment, the composition of the gut microbiome was altered. The relative abundances of Firmicutes and Bacteroidetes shifted closer to those of the DSS group. The phylum Proteobacteria has been reported as a prominent phylum of opportunistic pathogenic bacteria, contributing to the overproduction of pro-inflammatory cytokines and their association with the pathogenesis of IBD (35). Therefore, we hypothesize that the increase in inflammatory cytokines is primarily associated with the introduction of Pg rather than other pathogenic bacteria in the gut microbiota. At the genus level, S24-7 represents a predominant bacterial family within the mouse intestinal microbiota, although its taxonomic classification, sequence data annotation, and functional information remain limited. However, some research, such as the work by Rooks et al. (36), suggests that it may be associated with colitis remission. *Akkermansia*, an intestinal microorganism, which belongs to Verrucomicrobia, has been reported to relieve intestinal inflammation and promote intestinal epithelial healing. *Helicobacter* is a significant gastric pathogen, and research has indicated that *Helicobacter* infection can lead to dysbiosis in the intestinal microbiota, promoting an increase in harmful bacteria and a decrease in beneficial bacteria. This may potentially elevate the risk of IBD or colorectal cancer. The alterations in these bacterial genera align with the observed phenotypic changes. *Lactobacillus* has been found to promote the progression of gastric cancer by increasing reactive oxygen species and inducing immune tolerance (37). In our findings, the highest abundance of *Lactobacillus* was observed in the DSS + Pg group.

It has been indicated that both the microbiota and their metabolites affect the health of the host gut (38). Metabolites are small molecules that are produced as intermediate or end products of microbial metabolism (39). These metabolites can derive from bacterial metabolism of dietary substrates, modification of host molecules, such as bile acids, or directly from bacteria (40, 41). In our study, we employed untargeted metabolomics to elucidate the beneficial effects of paeoniflorin on gut metabolism. Our analysis revealed significant differences in four metabolic pathways: arginine biosynthesis, phenylalanine metabolism, galactose metabolism, and arachidonic acid metabolism. Spearman correlation analysis demonstrated a close relationship between these metabolites and various physiological indicators, highlighting their potential significance in the context of gut health. These pathways were significantly impacted, offering insights into the intricate metabolic changes that accompany inflammation.
